# Brown Adipose Tissue Energy Metabolism in Humans

**DOI:** 10.3389/fendo.2018.00447

**Published:** 2018-08-07

**Authors:** André C. Carpentier, Denis P. Blondin, Kirsi A. Virtanen, Denis Richard, François Haman, Éric E. Turcotte

**Affiliations:** ^1^Division of Endocrinology, Department of Medicine, Centre de Recherche du CHUS, Université de Sherbrooke, Sherbrooke, QC, Canada; ^2^Faculty of Medicine, University of Ottawa, Ottawa, ON, Canada; ^3^Turku PET Centre, Turku University Hospital, Turku, Finland; ^4^Institute of Public Health and Clinical Nutrition, University of Eastern Finland (UEF), Kuopio, Finland; ^5^Centre de Recherche de l'Institut Universitaire de Cardiologie et de Pneumologie de Québec, Université Laval, Quebec City, QC, Canada; ^6^Faculty of Health Sciences, University of Ottawa, Ottawa, ON, Canada; ^7^Department of Nuclear Medicine and Radiobiology, Centre de Recherche du CHUS, Université de Sherbrooke, Sherbrooke, QC, Canada

**Keywords:** brown adipose tissue, energy metabolism, obesity, type 2 diabetes, molecular imaging, positron emission tomography, tracer methods

## Abstract

The demonstration of metabolically active brown adipose tissue (BAT) in humans primarily using positron emission tomography coupled to computed tomography (PET/CT) with the glucose tracer 18-fluorodeoxyglucose (^18^FDG) has renewed the interest of the scientific and medical community in the possible role of BAT as a target for the prevention and treatment of obesity and type 2 diabetes (T2D). Here, we offer a comprehensive review of BAT energy metabolism in humans. Considerable advances in methods to measure BAT energy metabolism, including nonesterified fatty acids (NEFA), chylomicron-triglycerides (TG), oxygen, Krebs cycle rate, and intracellular TG have led to very good quantification of energy substrate metabolism *per* volume of active BAT *in vivo*. These studies have also shown that intracellular TG are likely the primary energy source of BAT upon activation by cold. Current estimates of BAT's contribution to energy expenditure range at the lower end of what would be potentially clinically relevant if chronically sustained. Yet, ^18^FDG PET/CT remains the gold-standard defining method to quantify total BAT volume of activity, used to calculate BAT's total energy expenditure. Unfortunately, BAT glucose metabolism better reflects BAT's insulin sensitivity and blood flow. It is now clear that most glucose taken up by BAT does not fuel mitochondrial oxidative metabolism and that BAT glucose uptake can therefore be disconnected from thermogenesis. Furthermore, BAT thermogenesis is efficiently recruited upon repeated cold exposure, doubling to tripling its total oxidative capacity, with reciprocal reduction of muscle thermogenesis. Recent data suggest that total BAT volume may be much larger than the typically observed 50–150 ml with ^18^FDG PET/CT. Therefore, the current estimates of total BAT thermogenesis, largely relying on total BAT volume using ^18^FDG PET/CT, may underestimate the true contribution of BAT to total energy expenditure. Quantification of the contribution of BAT to energy expenditure begs for the development of more integrated whole body *in vivo* methods.

## Introduction

Since 1980, the global prevalence of obesity has doubled (1). In 2015, overweight and obesity accounted for 4 million deaths worldwide, including 3.3 million from cardiovascular diseases and type 2 diabetes (T2D) (1). Restricting energy intake by reducing food consumption, increasing satiety and/or fat malabsorption, is the chief weight-loss mechanism of most medical and surgical treatments of obesity and has profound anti-diabetic effects (2–5). Increasing exercise- and non-exercise activity-related thermogenesis is the other cornerstone of obesity and T2D management. Simultaneously targeting multiple mechanisms of energy homeostasis is advantageous for the treatment of obesity (6). However, targeting energy expenditure unrelated to physical activity remains largely underexplored. Consequently, a number of unexploited mechanism may help fill a gap as an adjunct to current treatments for obesity and T2D.

One emerging, highly modifiable homeostatic mechanism for energy expenditure in humans is BAT thermogenesis. BAT may contribute as much as 60% of “non-shivering” thermogenesis in small mammals (7, 8), enabling their survival in the cold without reliance on shivering to produce heat (9, 10). BAT is currently considered a prime target for the treatment of obesity and T2D (11–15). Although the relative role of BAT on energy expenditure, thermogenesis and substrate utilization is dominant in rodents, the contribution of BAT to energy homeostasis in humans is more controversial. A detailed discussion on the different factors implicated in BAT and WAT “browning” such as immune cell-mediated modulation of adipose tissue sympathetic innervation (16) [please see (17) and (18) for review] is beyond the scope of the present review. The aim of the present article is to review the evidence for a role of BAT in energy substrate metabolism and thermogenesis in humans.

## The definition of bat

BAT is a heat-producing adipose tissue located in interscapular, subscapular, axillary, perirenal, and periaortic regions in rodents (19). In infants, the predominant interscapular distribution found in small mammals also occurs (20–22), but regresses with age and is lost at adulthood. The typical supraclavicular and paravertebral BAT distribution seen in adults appears to develop with puberty in boys and girls (23, 24). BAT cells differ from white adipose tissue (WAT) cells (25, 26). The former cells contain numerous small lipid vacuoles and a large number of well-developed mitochondria, whereas the latter are characterized by a single large lipid vacuole and a few mitochondria. BAT cells in WAT depots, called “beige” or “brite” adipocytes, have also been shown in rodents and humans (26). Histologically, “beige” cells demonstrate an intermediate phenotype between classical BAT adipocytes and classical white adipocytes (26).

The hallmark of BAT cells at the molecular level in animals and humans alike is the high level of expression of uncoupling protein-1 (UCP1). UCP1 is found in the inner membrane of BAT cells' mitochondria (19, 27). UCP1 uncouples mitochondrial respiration from adenosine-5′-triphosphate (ATP) synthesis (28). When activated, it causes a leak that dissipates the electrochemical proton gradient that builds up across the inner mitochondrial membrane during BAT fatty acid oxidation. This electrochemical proton gradient drives the conversion of adenosine-5′-diphosphate (ADP) to ATP by ATP synthase. As a consequence, the presence of active UCP1 abolishes the negative feedback inhibition exerted by high ATP and/or low ADP levels on mitochondrial Krebs' cycle and respiration, leading to very high rate of fatty acid oxidation that directly produces heat. Because of its large amount of active UCP1 proteins, BAT is thus the only organ that literally can “burn” fat.

**Figure 1:**
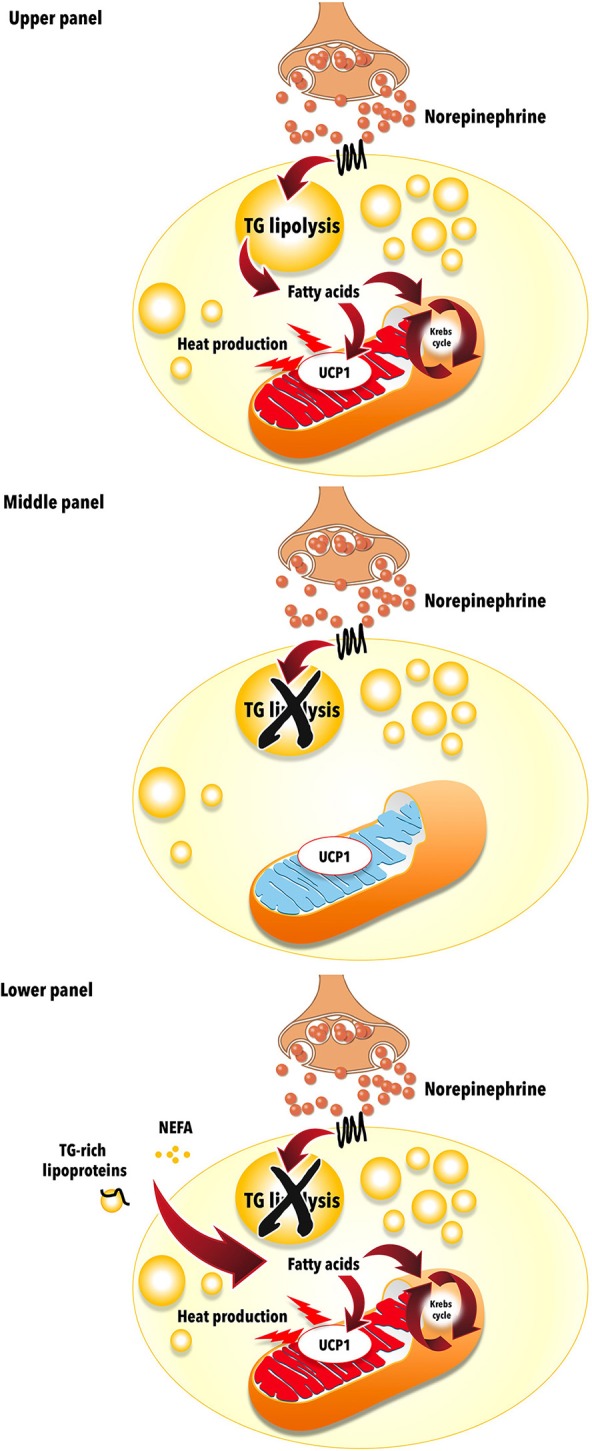
Uncoupling protein 1 (UCP1)-mediated brown adipose tissue thermogenesis. **Upper panel:** Brown adipose tissue UCP1-mediated thermogenesis is activated by fatty acids produced via norepinephrine-induced intracellular triglyceride (TG) lipolysis during cold exposure. **Middle panel:** Acute pharmacological inhibition of intracellular TG lipolysis blunts brown adipose tissue thermogenesis via reduction of intracellular fatty acids availability. **Lower panel:** Genetic deletion-mediated inhibition of intracellular TG lipolysis in brown adipose tissues leads to increased reliance on circulating nonesterified fatty acids (NEFA) and triglyceride (TG)-rich lipoproteins to sustain UCP1-mediated thermogenesis. The mitochondrion illustration was obtained free of copyright from Pixabay (www.pixabay.com, 2018).

UCP1 is activated by long chain fatty acids (19, 28, 29), but the mechanism by which it uncouples mitochondrial respiration has long been debated (30–32). UCP1 is an anion/H^+^ symporter that binds avidly long chain fatty acids, making it in effect a proton translocator (33). BAT is richly innervated by sympathetic nervous system efferent fibers and sympathetic activation is the physiological activator of BAT thermogenesis (19, 34–37). The release by these fibers of noradrenaline stimulates BAT intracellular triglyceride (TG) lipolysis, releasing long chain fatty acids that in turn activate UCP1 and BAT thermogenesis (Figure 1). We provided *in vivo* experimental evidence for this model by showing that nicotinic acid administration, an inhibitor of intracellular TG lipolysis, blocks acute cold-stimulated BAT thermogenesis in rats (38) and in humans (39). Recent investigations using genetic deletion of genes essential for intracellular TG lipolysis in mice models have, however, casted doubt about the essential role of intracellular TG lipolysis-derived fatty acids to activate BAT thermogenesis (40, 41). However, direct *in vivo* assessment of BAT thermogenesis was not measured and BAT of these genetic mouse models displayed a large increase in utilization of circulating fatty acids and glucose. It is therefore likely that intracellular TG and, if the later are unavailable circulating fatty acids, play an important role for the activation of BAT thermogenesis.

It is clear that BAT cells can stem from different cell lineages and display different molecular signatures depending on whether they are harvested from classical BAT or classical WAT depots (42–45). This molecular signature of supraclavicular BAT depots in humans may also be much more similar to that of “beige” adipocytes than that of “classical BAT” of rodents (46). Despite these differences, UCP1 content and function appear similar between human and mouse BAT (47). The distinct molecular signature of BAT could potentially be exploited for the *in vivo* identification and quantification of BAT. For example, targeting of a relatively BAT specific molecule, programed death ligand-1, was recently proposed for PET imaging and to quantify BAT in mice (48). However, from an integrative physiology and clinical perspective, it is the unique thermogenic potential of BAT, not its molecular signature, that matters. The presence of BAT in human adults has been noticed earlier from pathological investigations (49–51). Despite this early pathological description, the presence of functional BAT in adult humans was widely acknowledged only with the use of positron emission tomography coupled with computed tomography (PET/CT) with the glucose analogue 18-fluoro-deoxyglucose [^18^FDG) (52–57). It is the very intense metabolic activity of otherwise metabolically quiescent fat tissue, at least with regards to glucose metabolism, that led the scientific community to finally acknowledge BAT as an organ of interest for energy balance and as a potential therapeutic target for obesity and T2D.

**Figure 2:**
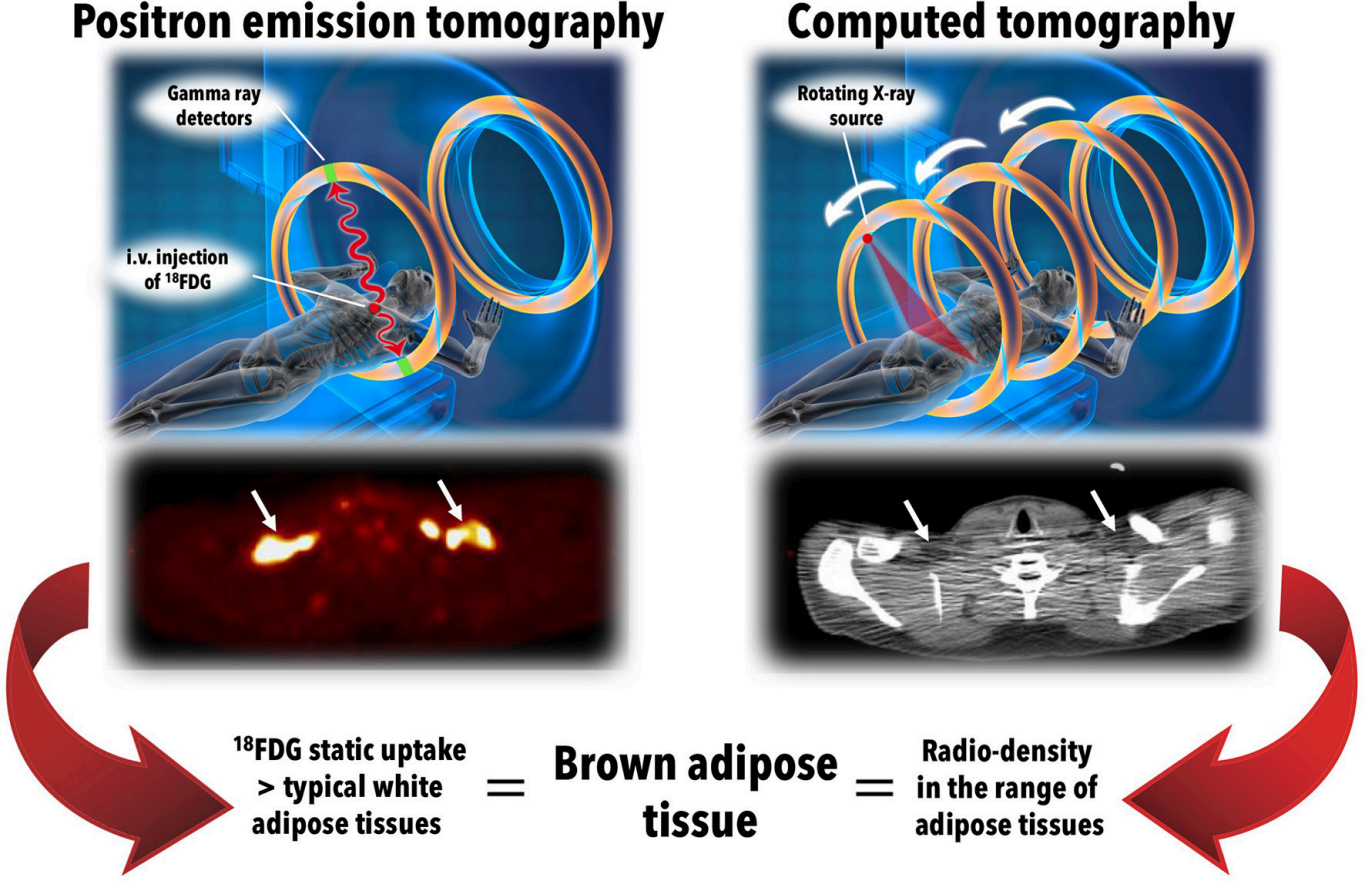
The standard definition of brown adipose tissue *in vivo* in humans. Brown adipose tissue is currently defined *in vivo* in humans by the combination of two radiological features: (1) 18-fluorodeoxyglucose (^18^FDG) uptake above a set threshold higher than that usually observed in white adipose tissues using positron emission tomography (**left panels**); and (2) a radio-density that is compatible with the presence of adipose tissue using computed tomography (**right panels**). After intravenous (i.v.) injection of ^18^FDG, whole body (static) positron emission tomography scanning is performed, giving quantitative tissue bio-distribution of the tracer into brown adipose tissues. This tissue tracer uptake is co-registered with tissue radio-density measured using computed tomography. The middle left and right panels show positron emission tomography and computed tomography transverse views, respectively, of supraclavicular brown adipose tissue in a healthy individual during a standardized cooling protocol. Source of illustration: Shutterstock (www.shuterstock.com, 2018, no. 100687138).

Currently, ^18^FDG PET/CT is considered the “gold-standard” method to identify BAT in humans (58), although BAT glucose metabolism does not accurately reflect BAT thermogenic activity (see section on glucose metabolism below) (59). The presence of BAT is defined according to the combination of two tissue characteristics on static (whole body) ^18^FDG PET/CT acquisition (Figure 2): (1) unusually high ^18^FDG (glucose) uptake for an adipose tissue, i.e., ^18^FDG PET standard uptake value normalized for lean mass higher than that of the upper range normally seen in classical WAT; and (2) a tissue radio-density on CT that is compatible with the presence of adipose tissue. Using ^18^FDG PET/CT, most of the glucose-utilizing BAT volume (“^18^FDG positive fat”) is constituted by multiple small adipose depots scattered in the supraclavicular, paravertebral, pericardial, and suprarenal regions (54, 56, 57, 60). Using ^18^FDG PET/CT, measured BAT volume in humans varies over two orders of magnitude, from a few to hundreds of milliliters (59). Three-dimensional mapping of adipose tissue depots with ^18^FDG PET/CT showed that up to 4.3% of total body adipose tissue mass accounts for depots that may display significant glucose uptake upon cold exposure (61). However, the proportion of this adipose tissue mass that was demonstrated as BAT mass using ^18^FDG PET/CT is very small, especially in obese individuals. It is important to note that accurate quantification of total BAT volume of metabolic activity by the addition of numerous small regions, typically less than 1 cm^3^ each, is very challenging using PET for a number of technical reasons that were discussed in more details elsewhere (59, 62). ^18^FDG positive fat sites are also determined by a series of environmental and biological factors including outdoor temperature preceding PET/CT scanning procedures, age, sex, body fat content, central adiposity, the presence of diabetes, circadian rhythm, and the use of some drugs such as β-adrenergic blockers (54, 55, 60, 63–70). The prevalence of spontaneously detectable ^18^FDG positive fat sites range from 2 to 7% in large cohorts of patients evaluated for cancer, but reaches 70–100% during experimental cold exposure (58, 59). ^18^FDG positive BAT volume and/or activity also significantly increases within weeks of cold acclimation (71–74). Glucose uptake in BAT is profoundly influenced by insulin sensitivity (see section on glucose metabolism below). Because of these technical and biological reasons, ^18^FDG PET/CT therefore likely underestimates true BAT volume in humans, especially in people with obesity and T2D.

Despite emerging methods using other PET tracers (48, 75–79), single-photon emission computed tomography (67, 80), magnetic resonance imaging (MRI), and spectroscopy (MRS) (81–89), near infrared spectroscopy (90, 91), contrast ultrasound (92), microwave radiometry (93), and optoacoustic imaging (94), ^18^FDG PET/CT currently remains the best method to define the presence and to measure BAT volume in humans (95–98) (99). The lack of a method that directly measure total BAT volume and BAT-specific thermogenesis, however, constitutes an important gap to fill in order to accurately define the true contribution of BAT to energy homeostasis in humans.

## Energy substrates utilization by bat

### Glucose

The demonstration of large increase in BAT glucose uptake with the activation of BAT oxidative metabolism led to the suggestion that BAT metabolic activation could be exploited to increase glucose clearance and utilization and treat diabetes (100, 101). This possibility was furthermore supported by recent epidemiological observations showing an association between increased glycosylated hemoglobin and increased incidence of diabetes with higher outdoor temperature (68, 102). Additionally, it was shown that the incidence of gestational diabetes rises by 6% for every 10°C increase in mean 30-day outdoor air temperature (103). Cold-induced whole body glucose disposal was shown to increase only in ^18^FDG BAT positive individuals (104) and BAT activation with cold exposure is furthermore associated with improved glucose homeostasis and insulin sensitivity in patients with T2D (105, 106).

**Figure 3:**
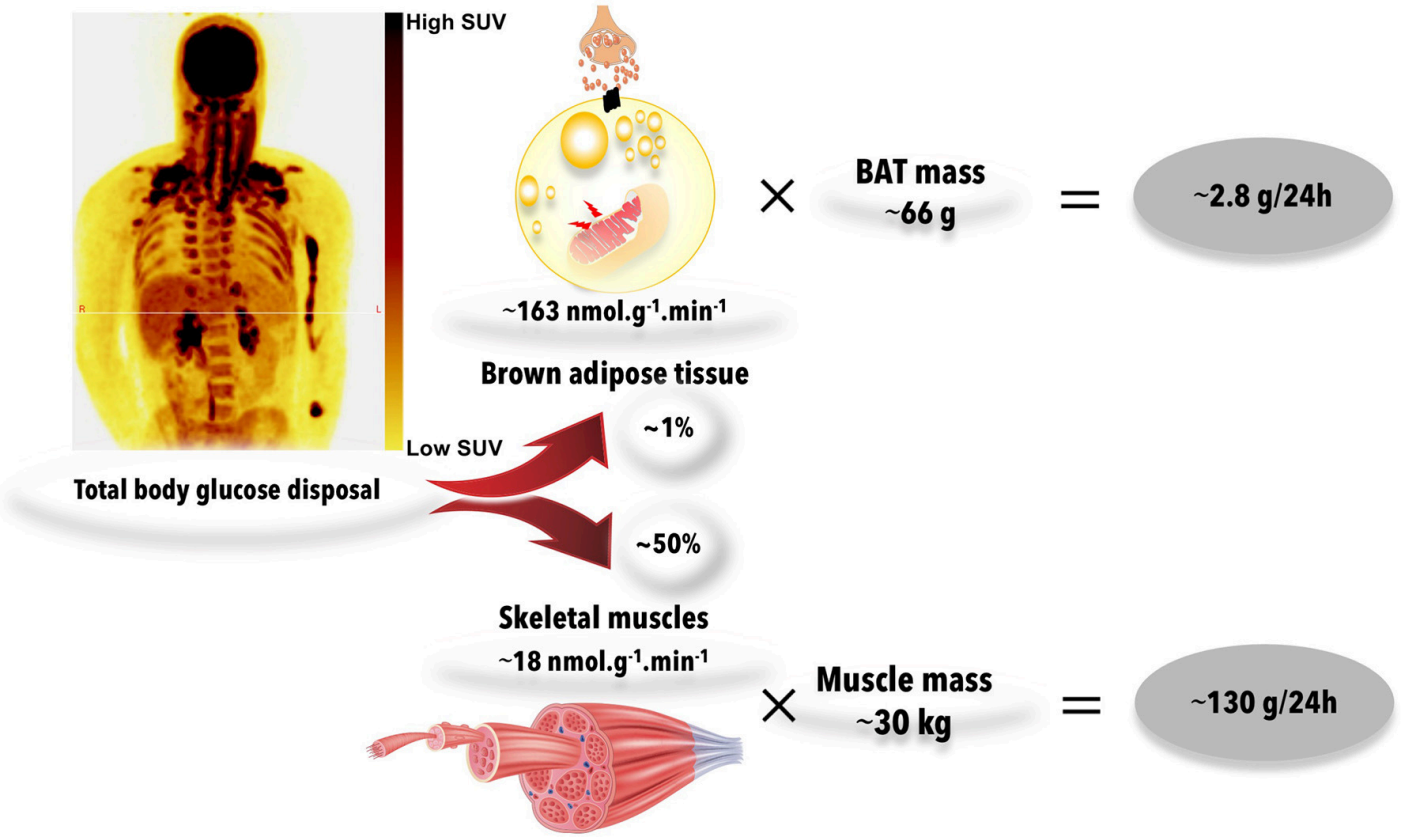
Whole body glucose uptake into brown adipose tissues and muscles during acute cold exposure. During mild cold exposure, glucose uptake is stimulated in brown adipose tissue, but also in several centrally-located skeletal muscles. Brown adipose tissue glucose uptake is ~8-fold higher than that of skeletal muscles, on average, per gram of tissue during mild cold exposure. However, total mass of brown adipose tissue is about 0.2% of that of skeletal muscles. Therefore, brown adipose tissue and skeletal muscle glucose uptake account for ~1 and 50%, respectively, of systemic glucose disposal. The figures presented were calculated from previously published data in young healthy individuals, before cold acclimation (39). BAT, brown adipose tissue; SUV, standard uptake value. Source of muscle illustration: Shutterstock (www.shuterstock.com, 2018, no. 404668558).

**Figure 4:**
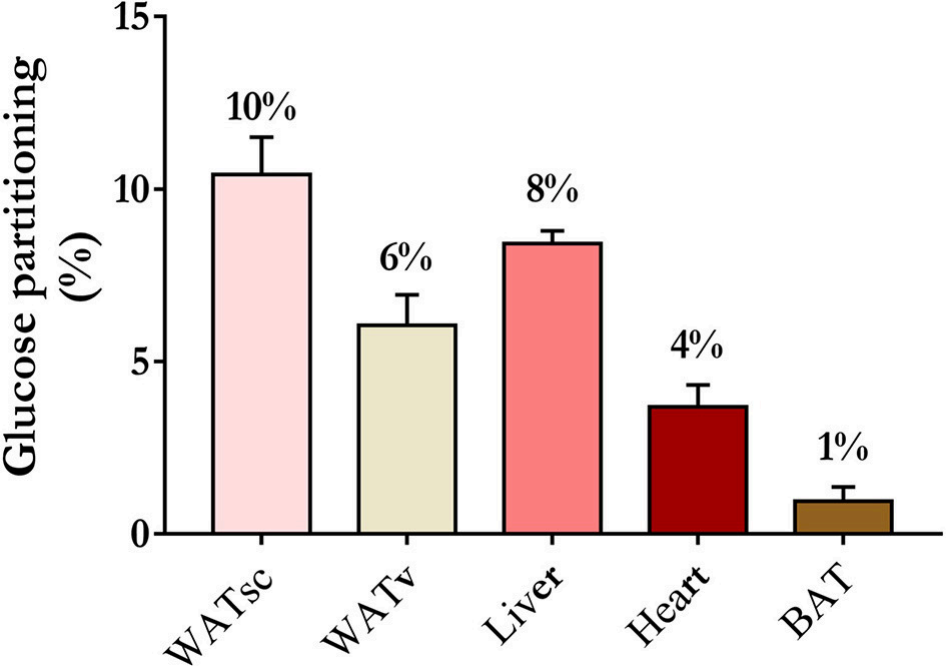
Organ-specific glucose partitioning during acute cold exposure. The figures presented were calculated from a previously published study in young healthy individuals, before cold acclimation (39), based on calculations that we detailed previously (110). BAT, brown adipose tissue; WATsc, sub-cutaneous white adipose tissues; WATv, visceral white adipose tissue.

There are, however, obvious problems with this hypothesis. First, cold exposure increases muscle *glut4* cell membrane expression and stimulates shivering and deep muscle glucose uptake, even when care is applied to limit muscle shivering (105, 107). Therefore, this muscle metabolic activity likely contributes to some cold-induced increase in whole body glucose disposal. Second, although cold-induced BAT glucose uptake *per* volume of tissue is indeed usually higher than that of other tissues in healthy subjects (107, 108), total volume of ^18^FDG-positive BAT amounts to <150 ml in most healthy individuals (59). ^18^FDG-positive BAT volume is also much smaller in individuals with obesity and T2D (109). This imposes an important limitation to the capacity of BAT metabolism to significantly impact systemic glucose clearance. For example, using whole body ^18^FDG PET acquisitions during standardized cold exposure in healthy subjects, we showed that BAT accounted for ~1% of total body glucose utilization as compared to ~50% for skeletal muscles (107) (Figure 3). Based on calculations that we previously described (110), glucose partitioning was 4, 8, 6, and 10% in the heart, liver, visceral WAT, and sub-cutaneous WAT, respectively (Figure 4).

**Figure 5:**
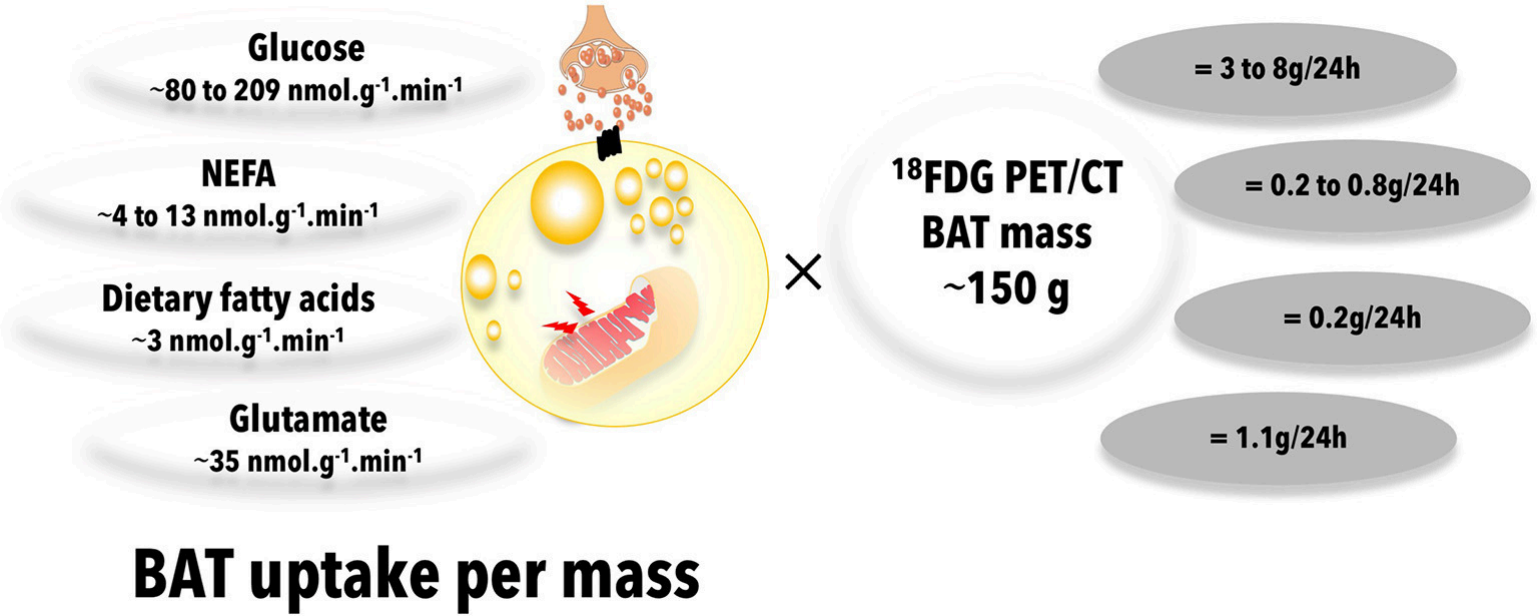
Brown adipose tissue uptake of energy substrates. Total brown adipose tissue uptake of energy substrates is calculated from published quantitative, dynamic positron emission tomography or microdialysis experiments in humans, multiplied by a typical total brown adipose tissue mass reported in the literature. Data from (73, 108, 113), (108, 113, 114), (115), and (116) were used to calculate glucose, NEFA, dietary fatty acid, and glutamate BAT uptake, respectively. ^18^FDG, 18-fluorodeoxyglucose; BAT, brown adipose tissue; NEFA, nonesterified fatty acids; PET/CT, positron emission tomography coupled with computed tomography.

Unfortunately, dynamic ^18^FDG PET acquisition allowing precise quantification of BAT glucose uptake rate has been used by only a few investigators. The group of University of Turku in Finland has reported BAT glucose uptake rates during acute cold exposure in the order of 90–120 nmol.g^−1^.min^−1^ in healthy individuals and of 35 nmol.g^−1^.min^−1^ in obese individuals (57, 111, 112). Our group at *Université de Sherbrooke* reported BAT glucose uptake rates during acute cold exposure at fasting ranging from 80 ± 14 nmol.g^−1^.min^−1^ in non-cold-acclimated healthy individuals to 209 ± 50 nmol.g^−1^.min^−1^ in cold-acclimated healthy individuals (39, 73, 108) (Figure 5 and Table 1). We found BAT glucose uptake during cold exposure in the postprandial period in the range of 50 nmol.g^−1^.min^−1^, i.e., not very different from those measured in the fasting state (115). Although these rates of glucose uptake are two to three-fold higher *per* volume of tissue than that measured in skeletal muscles, the much larger muscle vs. BAT mass translates into organ-specific uptake that is two orders of magnitude higher in the former (39). Furthermore, we found BAT glucose uptake rates to be much lower in older, overweight subjects without or with T2D, in the range of ~10 nmol.g^−1^.min^−1^ (109). In absolute terms, we found rates of BAT glucose uptake ranging from ~0.1 μmol/min in overweight individuals without and with T2D to ~3 μmol/min in healthy individuals during acute cold exposure. Using simultaneous quantification of BAT glucose uptake with dynamic ^18^FDG PET acquisition and systemic glucose utilization with conventional glucose tracer method, we found that acutely cold-activated BAT glucose uptake accounted for <1% of systemic glucose turnover in healthy men (39, 108, 109). It is therefore unlikely that BAT activation may significantly contribute to improve systemic glucose metabolism, especially in subjects with impaired glucose metabolism.

**Table 1:** Upper and lower estimates of brown adipose tissue plasma glucose, nonesterified fatty acid, dietary fat, and glutamate uptake rates in humans.

**Substrate**	**Mean uptake (nmol.g^−1^.min^−1^)**	**Molar mass (g.mol^−1^)**	**Absolute uptake assuming BAT mass of 150g (g.day^−1^)**	**Notes**	**References**
Glucose	80	180.156	3.11	Healthy men, non-cold acclimated, acute cold exposure	(39, 73, 108)
	209		8.13	Healthy men, post-cold acclimation, acute cold exposure	(73)
NEFA	4	275.446	0.24	Obese subjects, room temperature	(114)
	13		0.77	Healthy men, acute cold exposure	(108, 109)
Dietary fat	3	275.446	0.18	Healthy men, postprandial and acute cold exposure	(115)
Glutamate	35	147.13	1.11	Healthy men, acute cold exposure	(116)

*NEFA, nonesterified fatty acids*.

**Figure 6:**
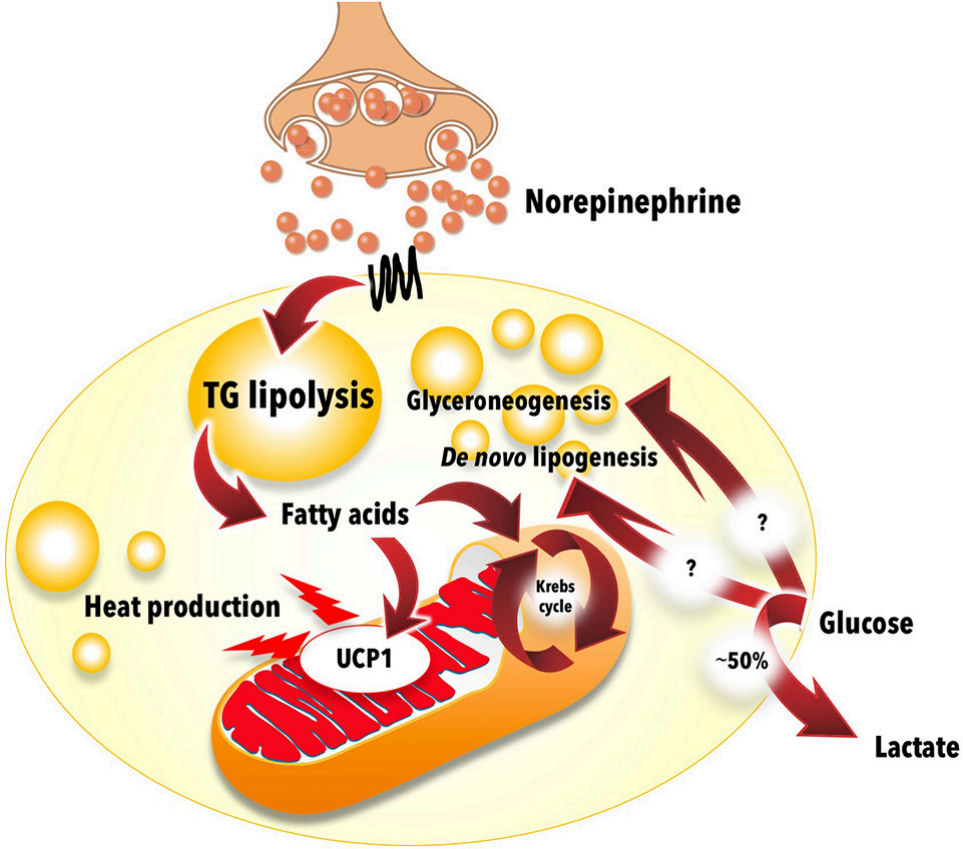
Glucose metabolism in brown adipose tissue. Most of the glucose taken up by brown adipose tissue during cold exposure does not contribute to thermogenesis. Experimental data show that approximately half of the glucose molecules are excreted from brown adipose tissue as lactate. Most of the remaining glucose likely contributes to glycerol production (glyceroneogenesis) and/or fatty acid synthesis (*de novo* lipogenesis) for intracellular triglyceride synthesis. The mitochondrion illustration was obtained free of copyright from Pixabay (www.pixabay.com, 2018).

BAT glucose uptake has been extensively used as a surrogate marker of BAT thermogenesis in humans on the basis of correlative observations between BAT thermogenic activity and glucose uptake. Indeed, the presence and metabolic activity of ^18^FDG positive BAT are associated with increased plasma catecholamines and inversely related to central obesity in patients with pheochromocytoma (117). Cold-induced BAT glucose uptake correlates with BAT sympathetic activity *in vivo* (118) and unilateral sympathetic denervation has been shown to reduce supraclavicular BAT glucose uptake in a patient (119). In mice however, β3-adrenergic-stimulated BAT glucose uptake does not need the presence of UCP1 and activation of BAT thermogenesis (120, 121). Extrapolated over a 24 h period, BAT glucose uptake in healthy individuals in our hands sums up only to a maximum utilization of 5 g of glucose, or ~23 kcal. Obviously, this energy expenditure rate assumes that BAT fully oxidizes the glucose it takes up. The classical studies by Ma and Foster (122), however, demonstrated more than three decades ago that a large fraction of glucose taken up by BAT is metabolized and released as lactate or serves for glyceroneogenesis (123) or perhaps *de novo* lipogenesis and does not contribute to increased BAT oxidative metabolism (Figure 6). Activated BAT glucose uptake exceeds increase in blood flow, suggesting non-thermogenic utilization of glucose by BAT in humans (124). A recent study using the adipose tissue microdialysis technique applied to supra-clavicular BAT also demonstrated that a large fraction of glucose taken up by BAT upon acute cold exposure is released as lactate *in vivo* in healthy subjects (116). The later study also independently confirmed the magnitude of glucose uptake in BAT measured by the ^18^FDG PET dynamic acquisition method. Thus, glucose uptake is not a good method to quantify BAT oxidative metabolism and thermogenesis, even in healthy subjects.

^18^FDG BAT positive individuals are more insulin sensitive and cold-induced BAT glucose uptake and stimulation of blood flow are blunted in obese individuals (112). BAT glucose uptake is reduced with genetic variants associated with insulin resistance (125), glucocorticoid treatment (126), fasting-induced insulin resistance (127). Chronic ephedrine administration which may induce insulin resistance leads to reduced BAT glucose uptake despite increased weight loss (128). BAT glucose uptake tends to be higher after bariatric surgery-induced weight loss in obese individuals (129, 130). Exercise, which increases muscle glucose uptake and improves whole body insulin sensitivity, does not however necessarily lead to increase in insulin-mediated BAT glucose uptake (131). Insulin stimulates BAT glucose uptake without stimulating blood flow, suggesting that insulin signaling increases BAT glucose uptake independent of BAT thermogenic activation (111). We found that older, overweight individuals without and with T2D display a ~10-fold reduction in BAT glucose uptake rate vs. young healthy subjects despite no reduction in BAT NEFA uptake and thermogenic activity upon acute cold exposure (109). Reduced BAT glucose uptake is furthermore associated with increased BAT fat content (88, 109, 132). Thus, as in lean tissues and WAT (133), excess lipid deposition appears to be a marker of BAT insulin resistance. Total BAT volume of ^18^FDG uptake has been associated with plasma NEFA appearance rate and oxidation and with WAT insulin sensitivity during cold exposure (109, 134). Thus, BAT glucose uptake may be a marker of WAT metabolic flexibility. In the presence of obesity, T2D or other insulin resistance states, BAT glucose uptake is an especially poor surrogate marker for BAT thermogenic activity.

Typical BAT cells expressing UCP1, large mitochondrial content and numerous small lipid vacuoles are present in supraclavicular adipose depots, independent of the presence of spontaneously active BAT based on ^18^FDG PET (135). Because ^18^FDG PET/CT is currently the only method capable of measuring BAT volume of metabolic activity, the above considerations clearly point to the absence of a reliable method to quantify BAT volume and, therefore, total thermogenic activity in humans. Unfortunately, all of the figures thus far reported with regards to the contribution of BAT to fatty acid utilization and whole body thermogenesis (see below) have been calculated using total BAT volume from ^18^FDG PET/CT. Therefore, these figures are likely underestimated, especially in subjects with any degree of insulin resistance.

### Circulating fatty acids

Utilization of circulating fatty acids by BAT may occur through two different pools in circulation: (1) NEFA; and (2) triglyceride-rich lipoproteins (TRL). Plasma NEFA are produced mostly by WAT, either via intracellular TG lipolysis or via LPL-mediated lipolysis of circulating TRL (i.e., NEFA spillover of TRL into the systemic circulation) (136). The circulating NEFA pool is tightly regulated by the sympathetic system and circulating insulin level via β-adrenergic stimulation and insulin signaling-mediated inhibition, respectively, of intracellular WAT lipolysis. Although plasma membrane fatty acid transporters (137) and local blood flow (138) are known to modify local tissue NEFA uptake, tissue NEFA transport rate is mostly regulated by the plasma NEFA concentration and by the tissue's rate of fatty acid oxidation. TRL include: (1) chylomicrons, produced by the intestine and transporting dietary fatty acids into the circulation; and (2) VLDL, produced by the liver and transporting TG from NEFA and lipoprotein-derived fatty acids recycled in the liver and fatty acids produced *de novo* from carbohydrates in the liver (110, 139). These two TRL circulating pools are mostly regulated through clearance mainly mediated by the activity of LPL, although increase in liver's VLDL-TG secretion rate also contributes to the increase of TG in circulation with obesity and T2D. Local tissue uptake of fatty acids from circulating TRL is mostly under the control of local tissue LPL-mediated lipolysis (140).

As can be expected from stimulation of the sympathetic system activity, acute cold exposure leads to robust increase in plasma NEFA levels and appearance rate (39, 108, 109, 141). Upregulation of genes of lipid utilization was shown in BAT with cold exposure in humans (134). Only a few studies however reported BAT-specific uptake rates of plasma NEFA. In all instances, this has been performed using the PET tracer ^18^F-fluoro-6-thiaheptadecanoic acid [^18^FTHA), a long-chain fatty acid analog that is taken up at similar rate than palmitate and that is trapped into the mitochondrial matrix and non-oxidative fatty acid metabolic pathways (142). These characteristics make this tracer, when administered intravenously, an excellent method to measure tissue-specific plasma NEFA uptake rate, but not tissue oxidative or non-oxidative metabolism. Using ^18^FTHA PET, BAT NEFA uptake was reported similar in healthy (~5.7 nmol.g^−1^.min^−1^) and obese subjects (~3.9 nmol.g^−1^.min^−1^, non-significant vs. healthy) at room temperature (Figure 5 and Table 1), only slightly higher than the NEFA uptake rate observed in subcutaneous neck WAT (~4.7 and ~3.4 nmol.g^−1^.min^−1^, respectively) (114). In the later study, slight but significant increase in BAT NEFA uptake rate was shown 6 months after bariatric surgery in obese individuals (~5.0 nmol.g^−1^.min^−1^) (114). Interestingly, BAT NEFA uptake was inversely correlated with age, waist circumference and percent body fat and directly correlated with HDL cholesterol level (114). Using ^18^FTHA PET, we reported BAT NEFA uptake rates ~13 nmol.g^−1^.min^−1^ in healthy young men acutely exposed to cold (108, 109) (Figure 5 and Table 1). We found BAT NEFA uptake in the same range as that observed in skeletal muscles and two to three-fold higher than that of subcutaneous WAT of the neck. In contrast to glucose uptake, BAT NEFA uptake *per* volume of tissue was the same in older, overweight participants without or with T2D compared to healthy young men (109). Because of the overlap in NEFA uptake between BAT and WAT and the limited experience with ^18^FTHA PET for BAT imaging, it has been thus far impossible to use this method to measure BAT volume, as performed using ^18^FDG PET/CT. Thus, current estimates of BAT total contribution to NEFA uptake is limited by the use of ^18^FDG PET/CT to measure BAT volume. Using the latter, we calculated that BAT may metabolize ~7 μmol.min^−1^ of plasma NEFA in healthy men exposed to cold, but only 0.1 μmol.min^−1^ in older overweight subjects without or with T2D (109). Extrapolated over a 24-h period, this amounts to up to 0.6 g of fat, or <3 kcal. Using simultaneous intravenous stable isotopic palmitate tracer, we calculated that BAT contribution to whole body NEFA metabolism is <1% (108, 109). Given the likely underestimation of BAT volume using ^18^FDG PET/CT, however, it is possible that BAT contribution to plasma NEFA metabolism could be higher. Cold-induced BAT NEFA uptake was shown to be associated with BAT thermogenesis (143). Therefore, the use of BAT NEFA uptake as a surrogate of BAT thermogenesis remains a viable alternative to glucose uptake. However, the use of PET NEFA tracers that can measure tissue oxidative and non-oxidative metabolic rates, as for example ^11^C-palmitate, will be needed to ensure that BAT NEFA uptake is quantitatively linked to BAT oxidative metabolism and not fatty acid esterification into BAT TG droplets.

Animal studies showing that activated BAT utilizes a large fraction of circulating TRL led to the hypothesis that active BAT may reduce circulating lipoprotein-TG and cholesterol in humans (144, 145). Angiopoietin-like 4 (ANGPTL4) is down-regulated in BAT during cold exposure in mice, leading to LPL-stimulated TG lipolysis and fatty acid uptake in BAT (146). Activated BAT in mice stimulates the formation of lipoprotein remnants from more buoyant TRL (147). Thus, in rodents, metabolically active BAT exerts significant impact on circulating TRL metabolism. Lower plasma TG and increased HDL-c has been observed in subjects with metabolically active BAT determined by ^18^FDG PET/CT (148). Experimental acute cold exposure in humans does not however lead to significant reduction in plasma TG levels (108, 109) and may even lead to small increase in TG and cholesterol levels in some instances (106, 141). To our knowledge, we published the only study that measured directly BAT uptake of fatty acids transported by TRL in humans (115). To achieve this, we used the oral ^18^FTHA PET method that we validated to measure organ-specific dietary fatty acid uptake (149). This method measures relative tissue uptake (partitioning) of dietary fatty acids from direct transport through chylomicron-TG and recycling from WAT metabolism as NEFA [see our recent review for a detailed discussion on the method (110)]. We demonstrated significant, albeit small, BAT dietary fatty acid uptake after administration of a standard meal during acute cold exposure in healthy young men (115). Rate of BAT dietary fatty acid uptake was calculated at ~3 nmol.g^−1^.min^−1^ (Figure 5 and Table 1), two to three-fold higher than in the neck subcutaneous WAT and skeletal muscles, respectively. Because of the small BAT volume, again determined using ^18^FDG PET/CT, BAT only contributed to 0.3% of whole body dietary fatty acid partitioning. In contrast to what has been observed in mice (144), BAT contribution's to whole body dietary fatty acid metabolism was much lower than that of the liver, the heart, skeletal muscles, and even WAT (115). Furthermore, we found that 4-week cold acclimation that significantly increased BAT oxidative metabolism in the participants did not increase BAT dietary fatty acid uptake (115). There was no relation between BAT oxidative metabolism and BAT dietary fatty acid uptake, suggesting that the latter is not a main energy substrate for BAT thermogenesis in humans, at least during acute cold exposure.

Cold-induced changes in plasma concentrations of some non-prominent fatty acids has been reported (106), but there was no demonstration that these changes were indeed due to increase in BAT metabolism. Cold exposure induces 12,13-dihydroxy-9Z-octadecenoic acid production in BAT and in circulation, which in turn may contribute to stimulate BAT fatty acid uptake in mice (150). In a cross-sectional study in healthy men, lysophosphatidylcholine-acyl C16:1 was shown to correlate with BAT volume and metabolic activity assessed by ^18^FDG PET/CT (151). The physiological and clinical relevance of these observations are unclear at the moment.

### Other substrates in circulation

BAT expresses glycerol kinase at higher levels than WAT and thus has the potential to utilize glycerol (152). Furthermore, this enzyme's expression is increased in BAT by cold exposure and β-adrenergic stimulation (153, 154). Recent experiments in mice showed that glycerol kinase is a downstream target of PPARγ and that its inhibition leads to reduced UCP1 expression, isoproterenol-stimulated cellular respiration and intracellular TG synthesis (155). A very recent study using adipose tissue microdialysis technique applied to supra-clavicular BAT in humans demonstrated reduced glycerol release by BAT vs. WAT at room temperature, suggesting that glycerol can be recycled to a greater extent in BAT compared to WAT (116).

Weir et al., using the microdialysis technique in supraclavicular BAT, reported significantly higher uptake of glutamate (i.e., ~35 nmol.g^−1^.min^−1^) by this tissue (Figure 5 and Table 1) vs. WAT (i.e., ~12 nmol.g^−1^.min^−1^) upon acute cold exposure (116). Uptake of glutamate in BAT, but not in WAT, was also significantly increased by acute cold exposure in the later study (by ~10 nmol.g^−1^.min^−1^), suggesting the use of this substrate for energy production or, alternatively, for anaplerosis. However, cold-induced increase in glucose uptake was about 10-fold (by ~120 nmol.g^−1^.min^−1^), showing that glutamate is a minor BAT substrate compared to glucose (116). Weir et al. also demonstrated net release of lactate (~150–200 nmol.g^−1^.min^−1^) and pyruvate (~5 nmol.g^−1^.min^−1^) by BAT that increased non significantly with acute cold exposure (by ~50 and ~1 nmol.g^−1^.min^−1^, respectively) (116). This release of lactate and pyruvate accounted for approximately half of the glucose that was taken up by BAT in response to cold.

To our knowledge, there has been thus far no attempt to quantify BAT utilization rate of ketones or amino acids *in vivo* in humans. Based on cardiac utilization rate of these substrates (156), it is however unlikely that they amount to a significant proportion of energy substrate utilization compared to fatty acids and glucose under most physiological circumstances.

### Intracellular triglycerides

Intracellular TG content of BAT can be quantified using CT or magnetic resonance imaging and spectroscopy (MRI/MRS). The former technique, as applied currently by most groups in the field of research on brown adipose tissue, is semi-quantitative and can only provide relative content of lipids in a tissue by comparing its radio-density (quantified in Hounsfield units). MRS is the gold-standard method for non-invasive quantification of tissue triglycerides (as opposed to total lipid content) and directly reports TG vs. water content of a tissue (62). BAT CT radio-density is strongly correlated with %TG by MRS (157). MRI can also provide quantitative fat-to-water ratios in BAT, that is lower than that observed in WAT (87). However, because of the large overlap observed in fat-to-water ratios, it is not possible to systematically distinguish metabolically active from non-active BAT or even WAT depots using quantification of adipose tissue fat fraction (158). These methods are sensitive enough to demonstrate association of fat fraction in BAT with biologically and clinically relevant end-points. For example, BAT fat fraction has been associated with systemic insulin resistance, central obesity or T2D (109, 114, 159) and with BAT NEFA uptake (157). BAT fat fraction is also reduced in obese individuals 6 months after bariatric surgery and this reduction is associated with reduction in BMI and insulin resistance (114).

**Figure 7:**
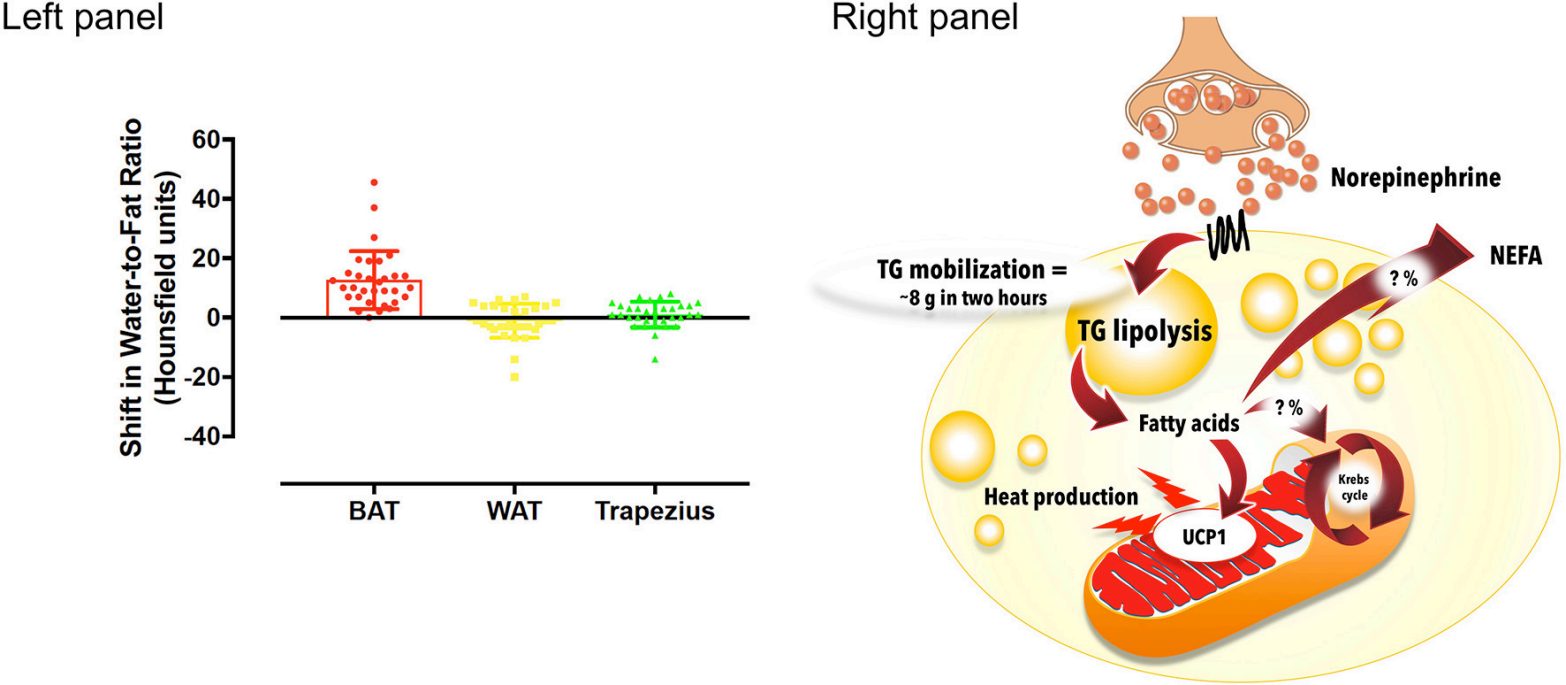
Intracellular triglyceride (TG) mobilization in brown adipose tissue during cold exposure. **Left panel:** Brown adipose tissue (BAT), white adipose tissue (WAT) and trapezius muscle change in radio-density during standardized acute cold exposure from previously published studies of our group (39, 73, 108, 109). **Right panel:** During cold-induced brown adipose tissue metabolic activation, up to 8 g of intracellular triglycerides can be mobilized within 2 h. The metabolic fate of the fatty acids that are mobilized is currently unknown. Although these fatty acids likely constitute most of the energy substrates driving brown adipose tissue thermogenesis, a fraction of them may also be released in circulation to be utilized by other tissues. NEFA, nonesterified fatty acids; UCP1, uncoupling protein 1. The mitochondrion illustration was obtained free of copyright from Pixabay (www.pixabay.com, 2018).

Early observation demonstrated that BAT radio-density increases rapidly during acute cold exposure in rats and humans (160). Numerous studies have now demonstrated that BAT TG content is hydrolyzed within 1–3 h through sympathetically-stimulated intracellular lipolysis, as observed using CT or MRI/MRS to monitor shifts of BAT water-to-fat ratio (73, 87, 108, 109, 115, 132, 158, 159, 161). This reduction in BAT TG content during acute cold exposure is specific to BAT and does not occur in WAT or in shivering muscles (Figure 7). It has also been related to whole body insulin sensitivity (132) and with plasma NEFA appearance rate (109). However, in contrast to cold-stimulated BAT glucose uptake, the rapid cold-induced reduction of BAT intracellular TG content is independent of age and T2D status, at equivalent cold exposure (109).

Assuming a total body BAT mass of 168 g [our ^18^FDG PET/CT data, (108)] and cold-induced reduction of BAT TG fraction from 81 to 76% (i.e., from 136 to 128 g of TG) (132), ~8 g of TG (~72 kcal) is mobilized from BAT over 2 hours of very mild cold exposure (Figure 7). *In vitro* experiments have suggested that up to 50% of fatty acids hydrolyzed by BAT could be released into the extracellular media (162) and subsequently oxidized or re-esterified elsewhere. It is therefore not possible to determine the precise contribution of fatty acids released by intracellular TG lipolysis to BAT thermogenesis currently, because the intracellular metabolic fate of fatty acids utilized by BAT has not yet been determined *in vivo* in humans. From BAT microdialysis data, glycerol release of BAT during cold exposure amounts to ~22 nmol.g^−1^.min^−1^ vs. ~10 nmol.g^−1^.min^−1^ at room temperature (116). It is not possible to directly measure tissue fatty acid release using microdialysis, but assuming that 3 fatty acid molecules are produced from intracellular TG *per* molecule of glycerol released, this cold-induced glycerol release (~12 nmol.g^−1^.min^−1^) could represent up to ~36 nmol.g^−1^.min^−1^ of fatty acids released by intracellular TG lipolysis in BAT. Assuming a fatty acid composition of ~30 palmitate, 30 linoleate, and 40% oleate (i.e., an average molar mass of 274.04 g.mol^−1^) (163), and an average BAT mass of 168 g (108), this would sum up to 2.4 g of fat over 24 h, or ~21 kcal. These figures, however, are likely underestimated given significant intracellular recycling of glycerol by BAT (116). Therefore, current estimates of fatty acid metabolism from BAT intracellular TG mobilization range between ~3 to up to 96 g over 24 h with sustained activation. Again, these figures depend on total BAT volume measured using ^18^FDG PET/CT and, therefore, are likely underestimated. It is also not known what proportion of these fatty acids are oxidized directly by BAT vs. released in circulation *in vivo* in humans.

We have shown in animals (38) and in humans (39) that BAT TG content is the primary source of energy that fuels BAT thermogenesis. We used nicotinic acid to inhibit intracellular BAT TG lipolysis *in vivo*, and to fully arrest BAT water-to-fat ratio shift and oxidative metabolism upon acute cold exposure. We also showed that blocking BAT thermogenesis with nicotinic acid reduced BAT glucose uptake by 26% (i.e., equivalent to 62 mg of glucose over the course of the study), with no change in systemic glucose turnover (39). This reduction in glucose uptake was most likely due to the abolished cold-induced increase in BAT oxidative metabolism with nicotinic acid, since fatty acids from intracellular TG activate UCP1-mediated mitochondrial energy uncoupling (19, 28, 29). Likewise, although not measured in our study, BAT NEFA uptake was likely also driven down by nicotinic acid-mediated inhibition of plasma NEFA appearance from WAT (39). Cold-stimulated BAT blood flow was unaffected by nicotinic acid, demonstrating that cold-induced water-to-fat ratio shift is indeed due to BAT TG disappearance, as opposed to increased blood flow. Importantly, muscle shivering rose reciprocally, compensating for the reduction in BAT thermogenesis. Given the small magnitude of the nicotinic acid-induced reduction of BAT glucose uptake and the currently estimated small contribution of plasma NEFA utilization by BAT in humans (see section Circulating fatty acids above), it is unlikely that these off-target effects of nicotinic acid confounded nicotinic acid effect through inhibition of intracellular TG lipolysis on BAT thermogenesis. In mice, gene deletion of key enzymes of BAT intracellular TG lipolysis induces a major increase in BAT utilization of fatty acids in circulation, thus substituting for BAT TG in order to sustain cold-induced thermogenesis (40, 41). In summary, these *in vivo* evidences suggest a major role for intracellular TG as fuel for BAT thermogenesis.

## Contribution of BAT to thermogenesis and energy expenditure

Some indirect evidences suggest a significant role for BAT in cold-induced thermogenesis in humans. Cold-induced increase in whole body energy expenditure is related to the presence of ^18^FDG positive BAT (164, 165). Cold-stimulated BAT blood flow is also associated with cold-induced whole body energy expenditure (111) and is blunted in obesity (112). Seasonal variation of cold-induced whole body energy expenditure is larger in ^18^FDG BAT positive subjects (166). Total ^18^FDG BAT volume is correlated with higher core body temperature during experimental cooling procedures in one study (167). Living in a mildly cold environment increases energy expenditure and, using ^18^FDG PET, BAT activation was shown to be a significant determinant of this response (168). We found that inhibition of BAT thermogenesis using nicotinic acid administration leads to reciprocal increase in muscle shivering to combat cold, suggesting a physiologically significant role for BAT in cold-induced thermogenesis (39). We also reported increased skeletal muscle energy coupling with cold acclimation—which is expected to reduce heat production at the same shivering intensity—suggesting an important role for BAT thermogenesis during cold acclimation (113).

Other indirect evidences suggest a role for BAT in energy expenditure and caloric balance in humans. The presence of metabolically active BAT assessed using ^18^FDG PET/CT is associated with reduced adiposity, especially with aging (56, 164, 169), with higher resting energy expenditure (56, 170), and with less ectopic fat deposition in the liver (171). Athletes, however, tend to have lower BAT volume and activity based on ^18^FDG PET/CT despite higher whole body energy expenditure (172, 173). UCP1 and beta-3 adrenergic receptor polymorphisms have been associated with lower BAT glucose metabolic activity and increased visceral fat with aging (174). Upstream stimulatory factor 1 deficiency that was shown to activate BAT metabolism in mice, is associated with improved insulin sensitivity, lipid profile and cardiometabolic risk in humans (175). Cold acclimation that increases BAT metabolic activity has been shown to reduce weight in some (72), but not all studies (73, 74, 104, 105, 176). The presence of ^18^FDG PET BAT predicts capsinoids, catechin-, and caffeine-stimulated whole body energy expenditure (177, 178). Treatment with capsinoids leads to increased BAT glucose uptake and supraclavicular temperature determined by near-infrared spectroscopy (90). Vagus nerve stimulation therapy associated with weight loss increases energy expenditure, which is associated with increased BAT glucose uptake (179). Ephedrine-stimulated BAT metabolic activity is blunted in obesity (180). However, other studies have found that Isoprenaline and ephedrine did not activate BAT metabolic activity despite increasing whole body energy expenditure in lean men (181, 182). Significant BAT contribution to energy expenditure is nevertheless supported by the β3-adrenergic agonist mirabegron-mediated increase in energy expenditure (+203 ± 40 kcal/day), associated with an increase in ^18^FDG BAT activity (183). However, this treatment is also associated with increased pulse rate and blood pressure, suggesting increased energy expenditure from the cardiovascular system.

Hypothyroidism and hyperthyroidism are conditions that reduce and increase, respectively, whole body energy expenditure. Although one study reported increased BAT glucose uptake with hyperthyroidism (184), others have reported no change in spontaneously occurring (185) or cold-induced BAT metabolic activity (186). Likewise, BAT activation has been observed with cancer cachexia (187, 188). Higher ^18^FDG BAT volume predicts less adipose tissue accumulation during cancer treatment in children (189). Association was also observed between reduction of BAT glucose uptake and chemotherapy-induced weight gain in women treated for breast cancer (190).

Some role for BAT in diet-induced thermogenesis has been proposed on the basis of preclinical studies (191). Postprandial increase in energy expenditure was reported to be higher in ^18^FDG BAT positive vs. BAT negative individuals, with lower respiratory quotient, but without significant change in total 24 h energy expenditure (192). BAT glucose uptake increases after meal intake, but is not related to diet-induced thermogenesis (193). Overfeeding, which increases energy expenditure (194), does not activate BAT glucose uptake (195). We showed no change in postprandial BAT dietary fatty acid and glucose uptake during cold exposure prior to vs. after cold acclimation for 4 weeks that activated BAT thermogenic activity 2 to 3-fold (115). This suggests that cold-induced BAT activation does not change organ-specific postprandial glucose or dietary fatty acid partitioning between organs. Interestingly, our study showed a non-statistically significant trend toward greater cold-induced increase in BAT radio-density postprandially after cold acclimation (115). A very recent study from the Turku group (196) demonstrated meal-induced BAT oxygen consumption equivalent to that observed with mild cold stimulation, together with significant reduction of BAT NEFA uptake and a trend toward higher BAT radio-density. Again, this suggests a more important role for intracellular TG vs. circulating substrates to fuel BAT thermogenesis in humans. The study of U Din et al. (196) estimated at ~13 kcal *per* day this meal-induced BAT contribution to energy expenditure. However, this calculation extrapolated BAT thermogenesis measured within the first postprandial hour to the entire day, which likely overestimates the contribution of this postprandial BAT thermogenesis to energy expenditure. Animal studies have consistently shown a decrease in classical BAT thermogenesis associated with a decrease in norepinephrine turnover with prolonged fasting (197). To our knowledge, there is no data available on the effect of prolonged or intermittent fasting on BAT activity or thermogenesis in humans.

The fact that BAT significantly takes up glucose, fatty acids, or other energy substrates from the circulation, and that it rapidly mobilizes its own TG content upon cold exposure does not prove that BAT contributes to thermogenesis and, therefore, to energy expenditure. Although direct BAT heat production was suggested by infrared spectroscopy [see (99) for review], this method cannot ascertain that heat difference measured on the surface of the skin overlying supraclavicular BAT is indeed produced by this organ. Likewise, measurement of BAT blood flow is an indirect measure of BAT thermogenesis and was shown in some instances to be dissociated from BAT thermogenesis (39). Supraclavicular BAT biopsies have been used to show higher *ex vivo* thermogenic activity in BAT vs. subcutaneous fat (134, 198). These biopsy methods are however incapable of measuring the true *in vivo* contribution of BAT to thermogenesis.

We used ^11^C-acetate, a tracer that allows quantification of Krebs' cycle rate through measure of ^11^CO_2_ BAT production with dynamic PET acquisition to demonstrate more direct evidence of BAT's contribution to cold-induced thermogenesis in humans (108). In the latter study, we found that cold-induced increase in tissue thermogenesis was observed in BAT, but not in neck WAT or skeletal muscles. Using this tracer, we found that BAT thermogenesis can be increased by 2 to 3-fold by acclimation to cold (73, 115), that it is not reduced in T2D vs. healthy individuals despite major reduction in BAT glucose uptake (109), and that it is blunted by inhibition of intracellular TG lipolysis with nicotinic acid (39). However, this method does not directly quantify BAT thermogenesis as ^11^CO_2_ tissue production is only a surrogate of tissue oxygen consumption.

Direct measurement of BAT oxygen consumption has been performed by the groups of Otto Muzik (199, 200) and that of the Turku PET Centre (143) using ^15^O_2_ dynamic PET acquisition. Using this method during very mild, short-term [60 min), but poorly controlled cold exposure, Muzik et al. estimated BAT thermogenesis to range between 15 to 25 kcal/day (200). The Turku group reported BAT thermogenesis figures in the range of ~7 kcal/day at room temperature to ~10 kcal/day during mild cold exposure in healthy subjects (143). Although BAT oxygen consumption *per* gram of tissue is 2 to 10-fold higher than that observed in WAT and skeletal muscles at room temperature or during mild cold exposure (143), the small BAT total tissue mass makes its relative contribution to basal and cold-induced thermogenesis very small. However, the small BAT total mass was again determined using ^18^FDG PET, which may lead to underestimation of the contribution of BAT to thermogenesis. Unfortunately, the current PET scanners with a limited field of view ranging from 16 to 24 cm in most instances do not allow total body dynamic acquisition during ^15^O_2_ or ^11^C-acetate administration. It is therefore not possible to simultaneously measure oxidative metabolism in all organs and all adipose tissue depots of the body. Furthermore, the very rapid tissue metabolism of these tracers makes impossible sequential dynamic acquisitions in different regions from the same tracer administration and safety considerations limit the number of sequential PET tracer administrations that can be made as part of experimental studies in humans. Therefore, the currently available methods cannot accurately determine total BAT contribution to thermogenesis.

**Figure 8:**
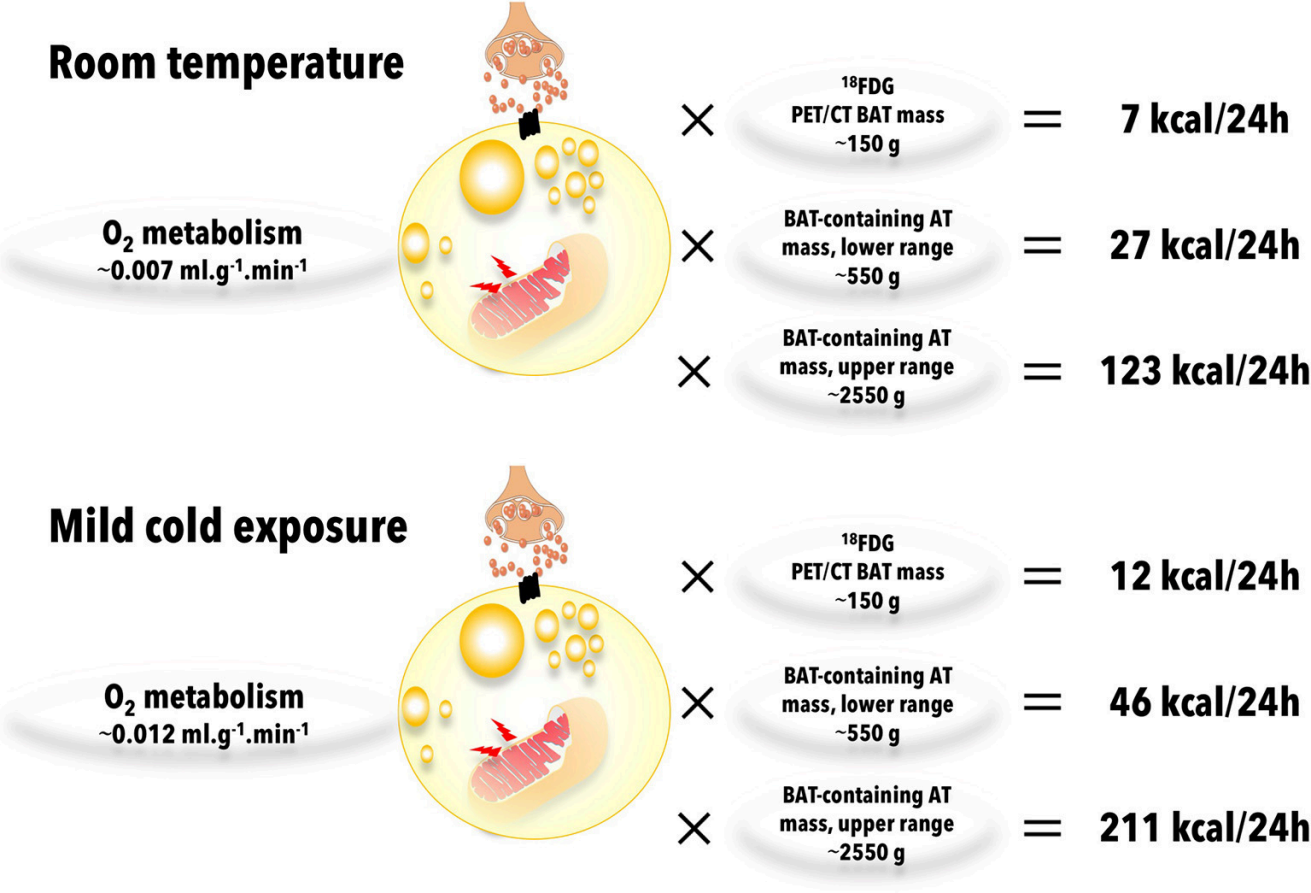
Brown adipose tissue (BAT) oxidative metabolism and contribution to total body energy expenditure. Brown adipose tissue oxygen consumptions are from U Din et al. (143) and brown adipose tissue-containing adipose tissue (AT) mass range is from Leitner et al. (61). Calculations were made assuming energy expenditure of 4.801 kcal *per* liter of oxygen consumed (201) and an adipose tissue density of 0.925 g.ml^−1^ (202). AT, adipose tissue.

Recently, radiological 3D mapping of possibly metabolically active adipose tissues has suggested a much greater metabolic potential for BAT (61). Using measures of total BAT volume from the later study (ranging from 510 to 2358 ml) (61) with the data on BAT oxidative metabolism *per* gram of tissue measured by U Din et al. (143) [0.007 ml.g^−1^.min^−1^ at room temperature and 0.012 ml.g^−1^.min^−1^ during cold exposure), and assuming energy expenditure of 4.801 kcal *per* liter of oxygen consumed (201) and an adipose tissue density of 0.925 g.ml^−1^ (202), BAT contribution to thermogenesis could range from 27–123 kcal *per* day at room temperature to 46–211 kcal *per* day during mild cold exposure (Figure 8). Accurate determination of oxidative metabolism over total body BAT volume will be critical to quantify the true potential of BAT in energy expenditure in humans.

## Is there a contribution of WAT browning to thermogenesis and energy expenditure?

In addition to the “classical” BAT depots, WAT “browning” (or “*beiging*”) may also contribute to thermogenesis, although this is still hotly debated (11, 203–206). A detailed discussion on the mechanisms and cellular adaptations of WAT “browning” is beyond the scope of this review and has been the subject of excellent recent papers (17, 207, 208). “Beige” cells express functional UCP1 and their development appears to be *Prdm16*-dependent, as classical BAT adipocytes (26, 44, 209). In rodents, chronic cold exposure, treatment with β_3_-adrenergic or PPARγ agonists, and exercise (38, 209–213) were shown to induce “browning” of WAT preferentially in subcutaneous depots, while reduced “browning” is seen with aging (214).

Although more controversial, there is also some evidence for physiologically significant WAT “browning” in humans. Ageing is associated with reduced white adipose tissue “browning” (169, 215, 216). Perirenal fat in women expresses UCP1 after exposure to cold environment, but difference in UCP1 expression does not translate into change in adipocyte respiration rate (217). Visceral fat “browning” *per* ^18^FDG PET/CT and histopathological examination has been shown in patients with pheochromocytoma or paraganglioma, associated with increased energy expenditure and diabetes (61, 218). Visceral fat glucose uptake was reduced by alpha blockade and removal of the tumor, associated with weight gain and reversal of diabetes. Cold acclimation however does not lead to “browning” of abdominal subcutaneous WAT in humans (71).

Seeing WAT “browning” using molecular markers, histological examination or even with ^18^FDG PET does not necessarily imply significant contribution to thermogenesis and energy expenditure. For example, PPARγ agonist treatment in rodents, while inducing WAT “browning” and BAT volume expansion, induces a reduction in sympathetic tone and thermogenesis (219–221). Recently, treatment with thiazolidinedione was shown to increase “browning” of subcutaneous WAT while reducing classical BAT glucose uptake and promoting weight gain *in vivo* in humans (222). We have shown in rats that chronic cold exposure or beta-3 adrenergic agonist treatment, while leading to robust “browning” of WAT as assessed by histological examination, UCP1 gene and protein expression, and mitochondrial DNA content, do not lead to significant increase in WAT thermogenic activity as assessed by ^11^C-acetate PET (213). This lack of WAT thermogenic activation contrasted with a very robust thermogenic activation of classical BAT simultaneously assessed by ^11^C-acetate PET in the same animals. It is also important to note that despite significant WAT “browning,” WAT UCP1 expression and mitochondrial DNA content remain one to two orders of magnitude below that observed in classical BAT (213, 223, 224). The total volume of WAT “browning” as well as its thermogenic activity have yet to be measured in humans. Based on the data available, it cannot be concluded at the moment that WAT “browning” plays a significant role in energy expenditure through mitochondrial uncoupling to similar extent as in “classical BAT” depots.

WAT “browning” can nevertheless potentially contribute to energy expenditure through activation of futile metabolic cycles. These cycles include TG lipolysis/esterification, activation of Na^+^-K^+^-ATPase, creatine/phosphocreatine cycling, and ATP-dependent Ca^2+^ cycling (206, 225, 226). For example, in the absence of UCP1, energy-consuming and heat-producing WAT metabolic adaptations nevertheless occur, including smaller multilocular lipid droplets, larger mitochondrial content, and increased sarcoendoplasmic reticulum Ca^2+^-ATPase expression (227). These adaptations are also associated with enhanced capacity of WAT to oxidize fatty acids (227), an outcome that can also be obtained using energy restriction or omega-3 supplementation (228). The group of Shingo Kajimura recently demonstrated UCP1-independent increased beige fat *ex vivo* glucose uptake, oxidation and thermogenesis mediated through enhanced ATP-dependent Ca^2+^ cycling from sarco/endoplasmic reticulum Ca^2+^-ATPase 2b and ryanodine receptor 2 (226). UCP1-independent BAT and beige fat thermogenesis from creatine/phosphocreatine cycling has also been demonstrated *in vitro* in human and murine cells and *in vivo* in mice (225).

During cold exposure, there is substantial activation of intracellular TG lipolysis in BAT and in WAT (see sections on Circulating fatty acids and Intracellular triglycerides above). Major stimulation of TG/fatty acid cycling is therefore expected to occur in both tissues, especially in BAT where glycerol can be utilized directly to a greater extent (116), and where glyceroneogenesis (123) and glycolysis (see section Glucose) occur at much faster rates. Of course, intracellular reesterification of fatty acids is fueled by Krebs' cycle and is accounted for when BAT oxidative metabolism is measured by the ^15^O_2_ or ^11^C-acetate PET methods. Nevertheless, part of the fatty acids produced by intracellular lipolysis in BAT could potentially be reesterified in other organs, leading to some additional energy expenditure. The extent of adipose tissue metabolic adaptation to prolonged cold exposure or pharmacological activation of BAT thermogenesis has not been determined thus far. However, adaptation of WAT TG/fatty acid cycling can reach impressive levels in humans. For example, using stable isotope tracer methodology in morbidly obese subjects before and 3 days, 3 months and 1 year after they underwent bariatric surgery, we showed that WAT NEFA production rate and storage capacity can be sustained at the same level despite a more than 3-fold reduction in adipose tissue mass (229), likely resulting in much enhanced energy expenditure *per* adipose tissue mass. Whether such WAT adaptations may contribute to chronic shift of total body energy balance remains to be tested.

## Clinical implications of BAT thermogenesis and energy substrate utilization

The obesity epidemic is mainly driven by a chronic positive energy balance—a difference of <0.1% between daily intake and expenditure—that is sustained over years. The average weight gain which when sustained over young adulthood leads to obesity by middle age is an incremental ~0.5–0.7 kg per year (230, 231). On a daily basis, this is an energy surplus of a mere ~12–17 kcal i.e., less than a 5 g cube of sugar, for an average energy density of 8840 kcal/kg of body (232). It is common among people who lose weight, to experience a drop in total energy expenditure—on average, ~25 kcal/day per kg of weight loss (232). This phenomenon, however, frustrates attempts by most people to maintain healthy weight. The inter-individual variability of this drop in energy expenditure can also significantly influence the rate of diet-induced weight loss (232, 233). When greater weight loss is maintained over time, the health advantages of lifestyle- and/or bariatric surgery-induced weight loss tend to be more significant (234–236). However, when as little as a ~2-kg weight loss is maintained over a span of 10 years—i.e., an average negative balance of ~48 kcal per day—the incidence of T2D is curbed by as much as 18–34% (237). Therefore, small shifts in energy balance that are sustained over time can exert major effects on health outcomes.

As discussed above, current estimates of BAT contribution to energy expenditure in humans are in the range of ~7–25 kcal *per* day based on ^15^O_2_ PET data recorded at room temperature or during mild acute cold exposure. Although small, these figures may be underestimated because of the current limitations of ^18^FDG PET to measure total BAT volume, especially in obese and T2D individuals. Whether enhanced TG/fatty acid recycling could add to BAT (or “beige” adipose tissue) thermogenesis has not been quantified in humans. It is also clear that BAT thermogenesis can be recruited to a significant extent with cold acclimation (73) and that its absence or stimulation lead to changes in cold-induced muscle shivering and non-shivering thermogenesis (39, 113). Activation of BAT thermogenesis may therefore be useful for people with occupational cold exposure. Attempts to activate BAT using cold over a prolonged period of time in humans did not result in weight loss in most (73, 74, 104, 105, 176) but not all studies (72), likely because of compensatory increase in energy intake. Circulating endocannabinoids increase with cold exposure (238), suggesting a possible mechanism for cold-induced increase in energy intake. It is also important to note that obese individuals need to reach lower skin temperature to induce cold-induced thermogenesis, due to increased body heat generation (i.e., increased resting energy expenditure driven by larger lean mass), not the generally falsely assumed increased fat layer insulation (239). The well-documented beneficial effect of chronic cold exposure on total body insulin sensitivity (74, 104, 105, 176) is driven by muscle, not BAT thermogenic activity (107). Therefore, cold-induced activation of BAT thermogenesis cannot by itself be proposed to shift caloric balance in humans. However, the possibility that it may serve as an adjunct preventive or therapeutic avenue to lifestyle interventions and/or appetite-suppressant drugs for obesity begs further investigations.

Rodent studies have suggested that BAT and/or WAT “browning” may be implicated in some of the beneficial metabolic effects of physical exercise [see (208, 240–242) for recent reviews on this topic]. At least four mechanisms have been evoked to drive such effects. First, physical exercise is associated with increased adrenergic stimulation, which may lead to direct activation of BAT thermogenesis and WAT “browning.” Second, exercise induces secretion of myokines such as irisin or meteorin like, cardiac natriuretic peptides, and fibroblast growth factor 21, that have all been implicated in BAT metabolic activity (243–245). Third, BAT-derived circulating factor such as IL-6 (246) or the recently discovered 12,13 diHOME (247) have been implicated in systemic metabolic improvement in mice. Fourth, exercise may lead to improved leptin and insulin signaling in the brain, with enhanced pro-opio-melanocortin neurons activation leading to WAT browning (241). However, exercise has long been shown to reduce cold-induced thermogenesis and cold acclimation in rats (248, 249), likely because heat produced by exercise down-regulates the sympathetic stimulatory output signal to BAT. As mentioned above, athletes have lower BAT volume and activity based on ^18^FDG PET/CT (172, 173) and BAT glucose uptake is reduced following short-term exercise training in healthy men (130). Different exercise duration, intensity and type can result in different adrenergic and systemic stress responses. Furthermore, as discussed above, BAT glucose uptake cannot be taken as a reliable index of BAT thermogenesis. To our knowledge, there is no study published in humans that has measured BAT thermogenesis *per se* in response to exercise. It is therefore difficult to draw definitive conclusions on the effect of exercise on BAT thermogenic activity in humans given the data currently available.

From epidemiological (retrospective) studies that have been published, there seems to be a graded South-to-North incremental prevalence of spontaneously active BAT. For example, the reported prevalence in Boston (latitude: 42°21′30″ North) is 4% (54) whereas it is 6.8% in Sherbrooke (latitude: 45°24′30″ North) (60). As it has been discussed above, cold acclimation leads to clear increase in BAT thermogenic activity and capacity in humans (73) and, therefore, cold exposure is an important driver of this South-to-North incremental prevalence of metabolically active BAT. Whether ethnic background differences may explain changes in BAT metabolic activity is more controversial. For example, direct comparison between subjects of South Asian vs. Europid descent showed either no change in BAT ^18^FDG uptake (250), or reduced BAT ^18^FDG volume (but not activity) (170). In the latter study, all participants were Dutch (therefore living at the same latitude) and participants from South Asian descent also displayed lower resting energy expenditure and lower cold-induced non-shivering thermogenesis. The same research group later demonstrated that this blunted thermogenic response in subjects from South Asian descent may be due to increased endocannabinoid tone (238). From the limited data available, we can conclude that cold exposure is clearly linked to differences of BAT activity between different populations, and that genetic/ethnic background differences may also play a role in modulating BAT metabolic responses. More data are needed to address this issue.

Despite encouraging results of BAT metabolic activation with a single-dose administration of mirabegron, a beta-3 adrenergic agonist (183), this class of agents has proven ineffective for the treatment of obesity or T2D in early clinical trials (251, 252). Furthermore, cardiovascular safety is of concern given the increase in heart rate and blood pressure observed with this class of medication, likely mediated through beta-1 and/or beta-2 spillover effects. Capsinoids, catechins and caffeine activate BAT glucose uptake and whole body energy expenditure (177, 178), but with variable results on clinical outcomes (253–256). The effect of beta-3 adrenergic agonists, capsinoids, and catechins on BAT thermogenesis however remains to be determined in humans. The intriguing possibility that BAT thermogenesis could be used as a *personalized medicine* approach to guide the use of these agents for the prevention or treatment of obesity also remains unexplored.

## Conclusion

BAT is a fascinating organ that possesses a very large thermogenic potential *per* mass of tissue. This tissue has an astounding capacity to rapidly mobilize its own TG content upon cold exposure. Fatty acids from this intense intracellular lipolysis are likely the main substrates for BAT thermogenesis, although the metabolic fate of fatty acids in BAT *in vivo* in humans has not yet been reported. The contribution of BAT and “beige” adipose tissue to thermogenesis through accelerated TG/fatty acid cycling also need to be quantified. BAT is of clear physiological relevance for cold-induced thermogenesis and is integral to a multisystem adaptive response to cold. The current estimated contribution of BAT to energy expenditure is however low due to its small volume measured using ^18^FDG PET. The latter method has major limitations for accurate measurement of BAT volume, likely leading to underestimation of the true contribution of this tissue to thermogenesis, especially in individuals with obesity and T2D. The current estimates of BAT thermogenesis are at the lower end of energy expenditure shifts that could lead to clinical benefits if sustained without off-target side-effects over the long term. Currently estimated plasma glucose, NEFA or lipoprotein utilization by BAT is also too low to be deemed of clinical relevance to treat T2D or lipid disorders. The development of novel imaging methods for accurate quantification of BAT volume is however required to delineate the true potential of targeting BAT thermogenesis to prevent and/or treat cardiometabolic disorders. With the demonstration of a slightly higher contribution to thermogenesis, it is still possible that metabolic activation of BAT could serve as an effective adjunct therapeutic target to existing treatments for obesity and T2D. Whether monitoring of the effect of clinical interventions on BAT thermogenesis may help personalize treatment selection for obesity and/or T2D also needs to be addressed in future studies.

## Author contributions

AC wrote the first draft of the manuscript and drafted all figures, except Figure 4. DB critically reviewed the manuscript, drafted Figure 4, and contributed to draft Figures 3, 7. KV, DR, FH, and ÉT critically reviewed the manuscript and contributed to draft Figure 2.

### Conflict of interest statement

The GSK Chair in Diabetes of the *Université de Sherbrooke* held by AC has been created in part through a donation of $1 million by GSK to the *Université de Sherbrooke*. AC has been recently funded by Janssen Canada to perform an investigator-initiated trial on the effect of treatment with canagliflozin on cardiac energy metabolism and function. AC has no other disclosures related to the content of this manuscript. AC holds research funding from the Canadian Institutes of Health Research, Canadian Diabetes Association, *Fonds de recherche Québec—Santé*, GSK, Janssen, Merck, Pfizer, AstraZeneca, Aventis, NovoNordisk, Eli Lilly, UniQure, Caprion Biosciences. AC has participated in advisory boards for the companies UniQure, Merck, Janssen and AstraZeneca and made one conference sponsored by AstraZeneca. The research and work at the basis of the present manuscript were not supported by any of these sources, aside from the GSK Chair in Diabetes of the *Université de Sherbrooke*. The remaining authors declare that the research was conducted in the absence of any commercial or financial relationships that could be construed as a potential conflict of interest.
